# Supplementary material for: Membrane-induced 2D phase separation of the focal adhesion protein talin
Source: Nat Commun. 2024 Jun 11;15:4986. doi: 10.1038/s41467-024-49222-z (PMC11166923; doi:10.1038/s41467-024-49222-z)
Supplement: Supplementary file 1 — Supplementary Information [file 41467_2024_49222_MOESM1_ESM.pdf]

Supplementary Figures

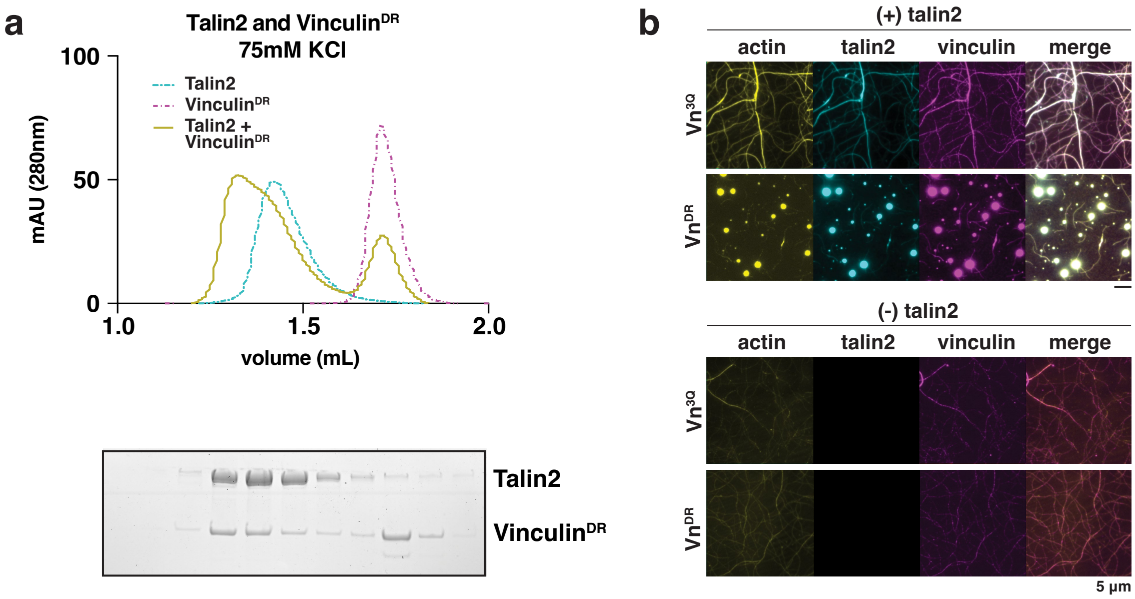

Supplementary Figure S1. Vn^{DR} can bind to Tn2 in low salt conditions, and requires Tn2 for phase separation. a Tn2 and Vn^{DR} (both 5 µM) reconstitution assay using size-exclusion chromatography (SEC). Chromatograms and SDS-PAGE indicate the elution profiles of the proteins alone and in combination for Tn2 and Vn^{DR} in 20 mM HEPES pH 7.8, 75 mM KCl, 1 mM EDTA, 3 mM β-mercaptoethanol. **b** The Vn^{3Q} mutation disrupts one of two vinculin head-tail autoinhibitory interactions, while Vn^{DR} has mutations at both autoinhibitory interfaces. Representative three-color TIRF microscopy images of 1.5 µM SNAP-tag-labeled FA proteins and 1 µM actin (10% actin-ATTO488) added to droplet buffer (10 mM imidazole, 50 mM KCl, 1 mM $MgCl_2$, 1 mM EGTA, 0.2 mM ATP, pH 7.5) supplemented with 15 mM glucose, 20 µg/mL catalase, 100 µg/mL glucose oxidase, 1 mM DTT and 0.25% methyl-cellulose (4000 cp). Images acquired after 30 min of actin polymerization. Vn^{3Q} and Vn^{DR} do not show obvious differences when incubated with actin alone, but in the presence of Tn, Vn^{DR} results in droplets while Vn^{3Q} results in actin bundling. This suggests that both autoinhibitory interactions between the vinculin head and tail domains must be released to trigger phase separation with talin. Scale bar is 5 µm.

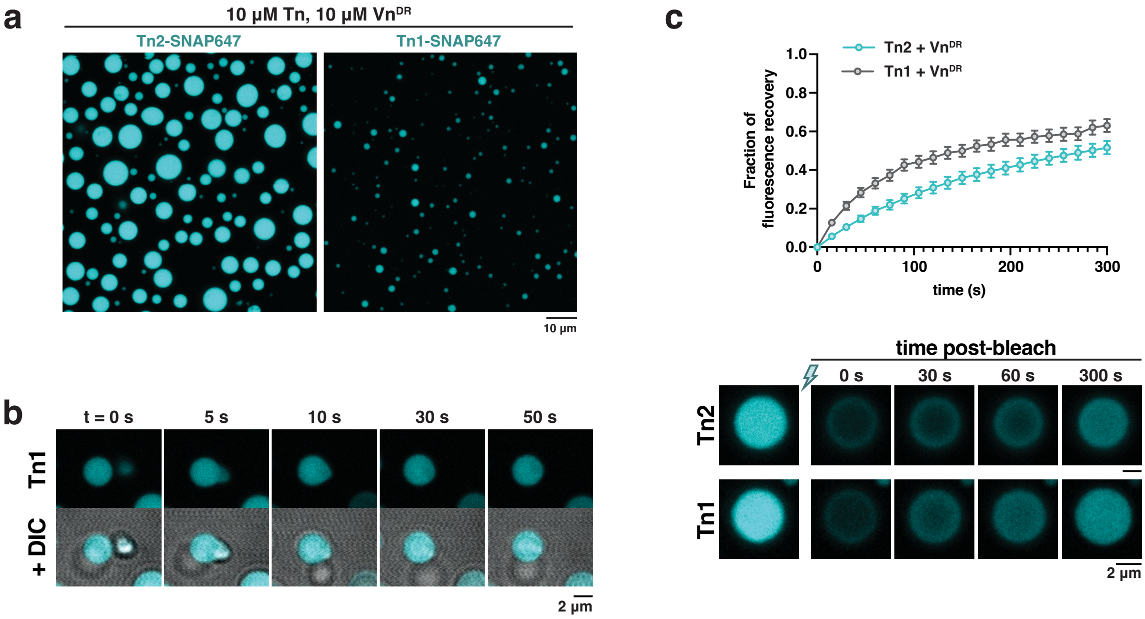

Supplementary Figure S2. Talin1 requires higher concentrations to phase separate with Vn^{DR}. a Representative image of Tn2-SNAP and Tn1-SNAP (10 µM) droplets that form upon mixing with Vn^{DR} (10 µM) in the presence of a crowding agent. Each sample was done in triplicate, and incubated for one hour before imaging. All samples were mixed in our droplet buffer (10 mM imidazole, 50 mM KCl, 1 mM $MgCl_2$, 1 mM EGTA, 0.2 mM ATP, pH 7.5) supplemented with 15 mM glucose, 20 µg/mL catalase, 100 µg/mL glucose oxidase, 1 mM DTT and 0.25% methyl-cellulose (4000 cp). **b** Tn1-Vn^{DR} droplets have liquid-like properties, and return to a spherical shape after fusing. **c** Fluorescence recovery after photobleaching of Tn1-Vn^{DR} and Tn2-Vn^{DR} droplets. Bleaching was carried out 15-30 minutes after initial droplet formation. Error bars represent standard error; n = 12 droplets for Tn1,Vn^{DR}, n= 6 droplets for Tn2,Vn^{DR}. Scale bars are indicated for each figure panel.

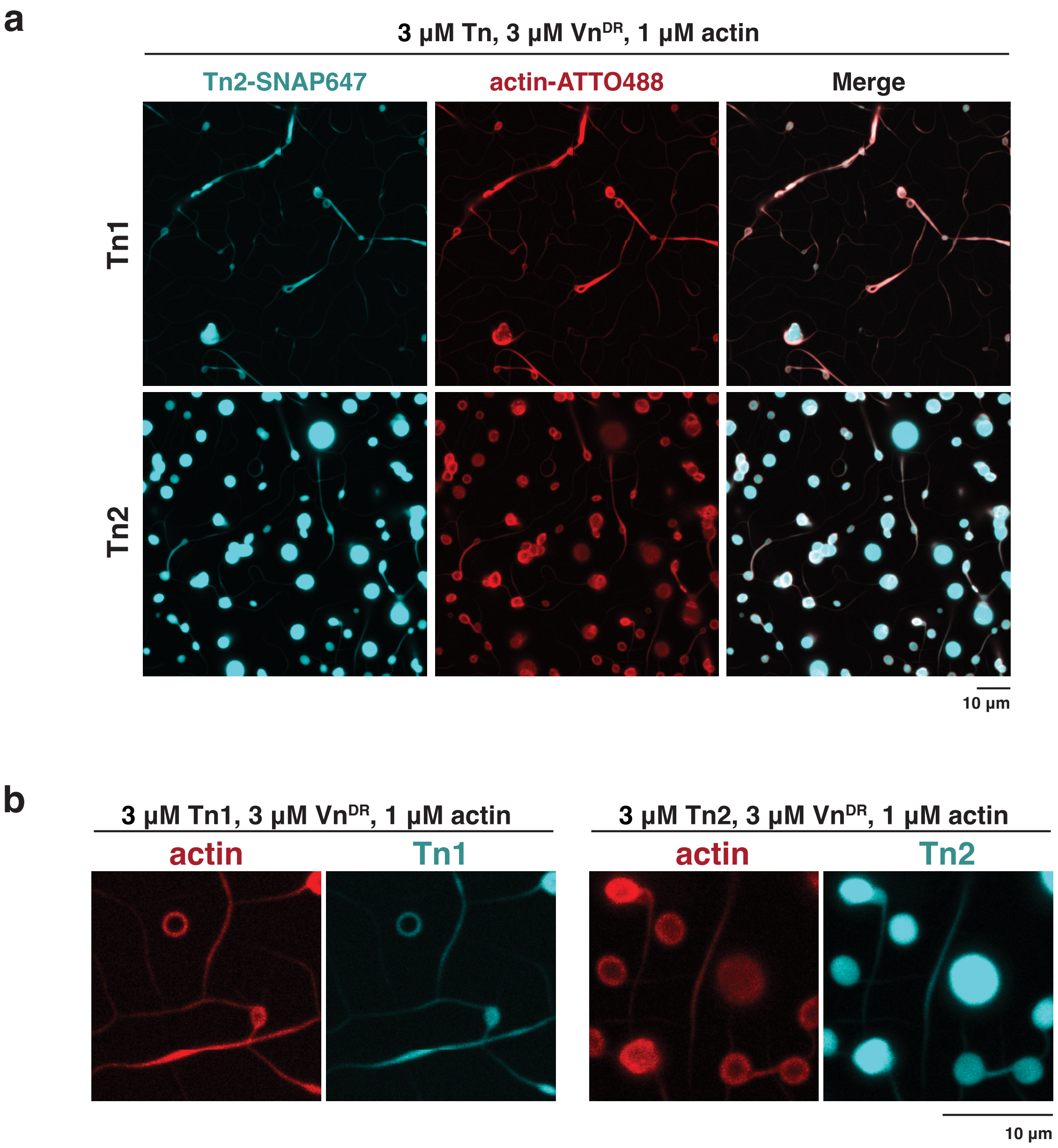

Supplementary Figure S3. Talin2 forms more droplets in the presence of actin than talin1. Representative three-color TIRF microscopy images of 3 μM SNAP-tag-labeled FA proteins and 1 μM G-actin (10% actin-ATTO488) in our droplet buffer (10 mM imidazole, 50 mM KCl, 1 mM $MgCl_2$, 1 mM EGTA, 0.2 mM ATP, pH 7.5) supplemented with 15 mM glucose, 20 μg/mL catalase, 100 μg/mL glucose oxidase, 1 mM DTT and 0.25% methyl-cellulose (4000 cp). Images acquired after 30 min of polymerization. **a** Tn1 with Vn^{DR} bundles actin filaments, occasionally forming droplet-like spots. Tn2 with Vn^{DR} forms actin-containing droplets, sometimes connected to actin bundles. **b** Zoomed-in view of Tn-Vn^{DR}-actin structures. Experiment done in triplicate. Scale bars are 10 μm.

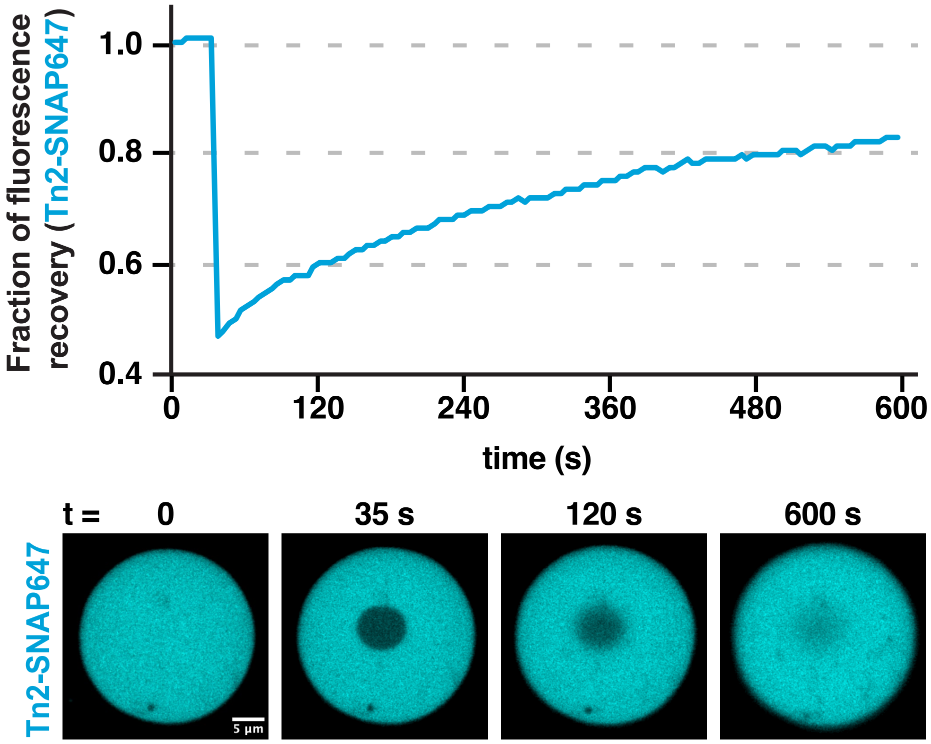

Supplementary Figure S4. FRAP experiment within Tn2 condensate. Fluorescence recovery after photobleaching a region within a large Tn2 condensate. Experiment with 2 µM Tn2, 2 µM Vn^{DR}, 0.25% methyl cellulose. Fluorescence in the bleached region reaches ~67% of original intensity after 10 min. The boundary between bleached and unbleached area becomes gradually more blurry over time, i.e. the bleached area recovers from the outside in. This suggests a diffusion-mediated process and thus indicates the liquid nature of the protein condensates. Shown is data from a single FRAP experiment.

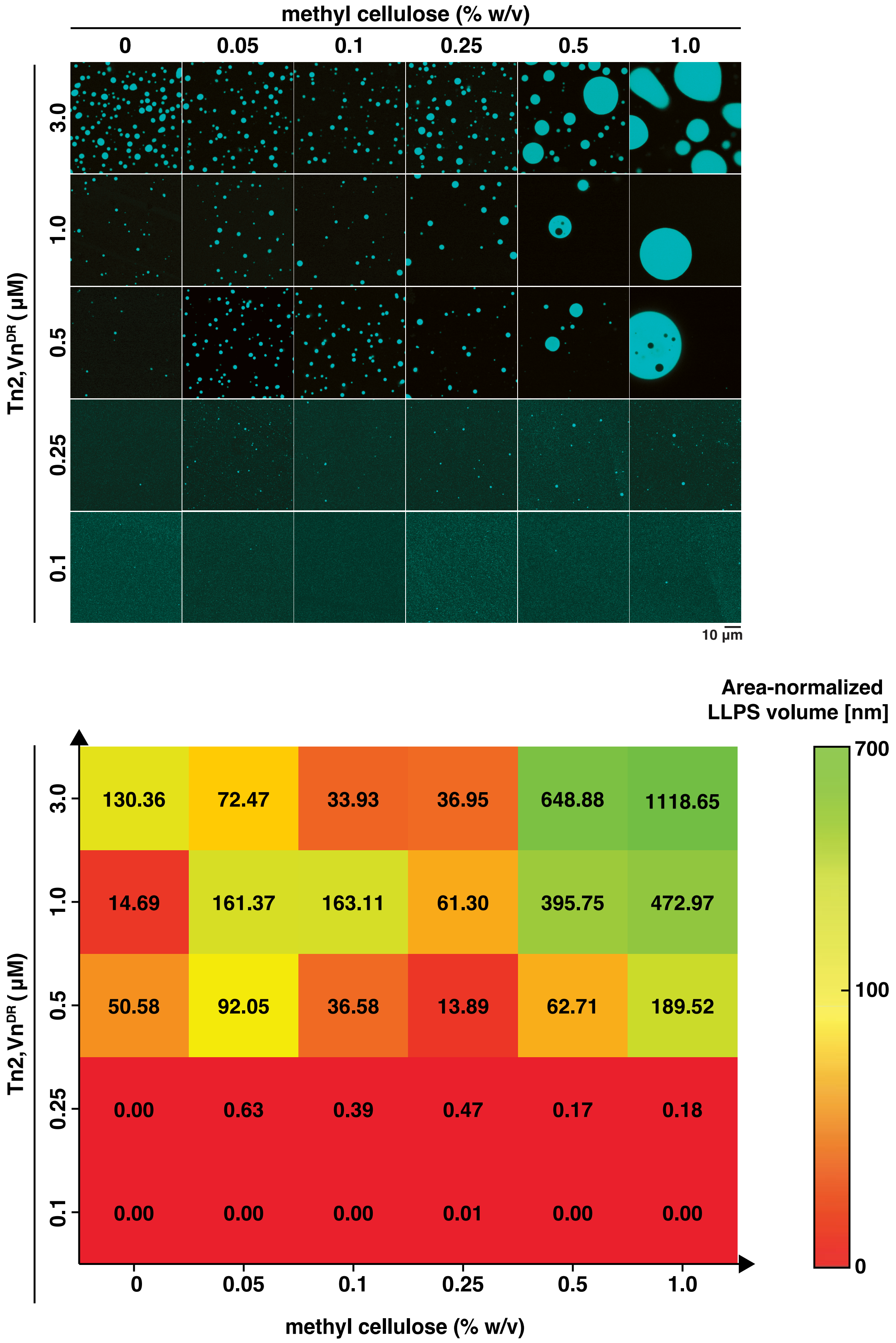

Supplementary Figure S5. Phase separation of talin is dependent on protein and crowding agent concentration. Representative images of droplet formation for

varying concentrations of Tn2 and Vn^{DR}, with varying amounts of crowding agent (methyl cellulose – 4000 cp). These experiments were carried out in a 384-well plate, with slightly different results from those observed in channel-slides. Still, the dependence on concentration is consistent with all other experiments, though the threshold for droplet formation varies between imaging set ups. Heat map shows mean values of quantification of area-normalized LLPS droplet volumes. For each conditions for n ≥ 6 regions with the samle were analyzed from one sample per condition. Scale bar is 10 μm.

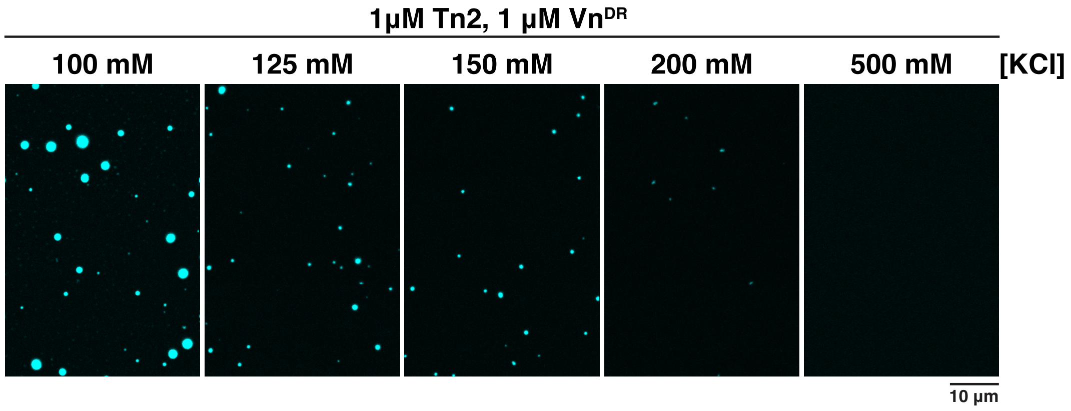

Supplementary Figure S6. Phase separation of talin is salt sensitive. The amount of phase separated material decreases with increasing amount of salt. Above 150 mM KCl, very few droplets are observed. Experiment done in triplicate, each sample was incubated for one hour in the following buffer (10 mM imidazole, X mM KCl, 1 mM $MgCl_2$, 1 mM EGTA, 0.2 mM ATP, pH 7.5) before imaging. Scale bar is 10 μm.

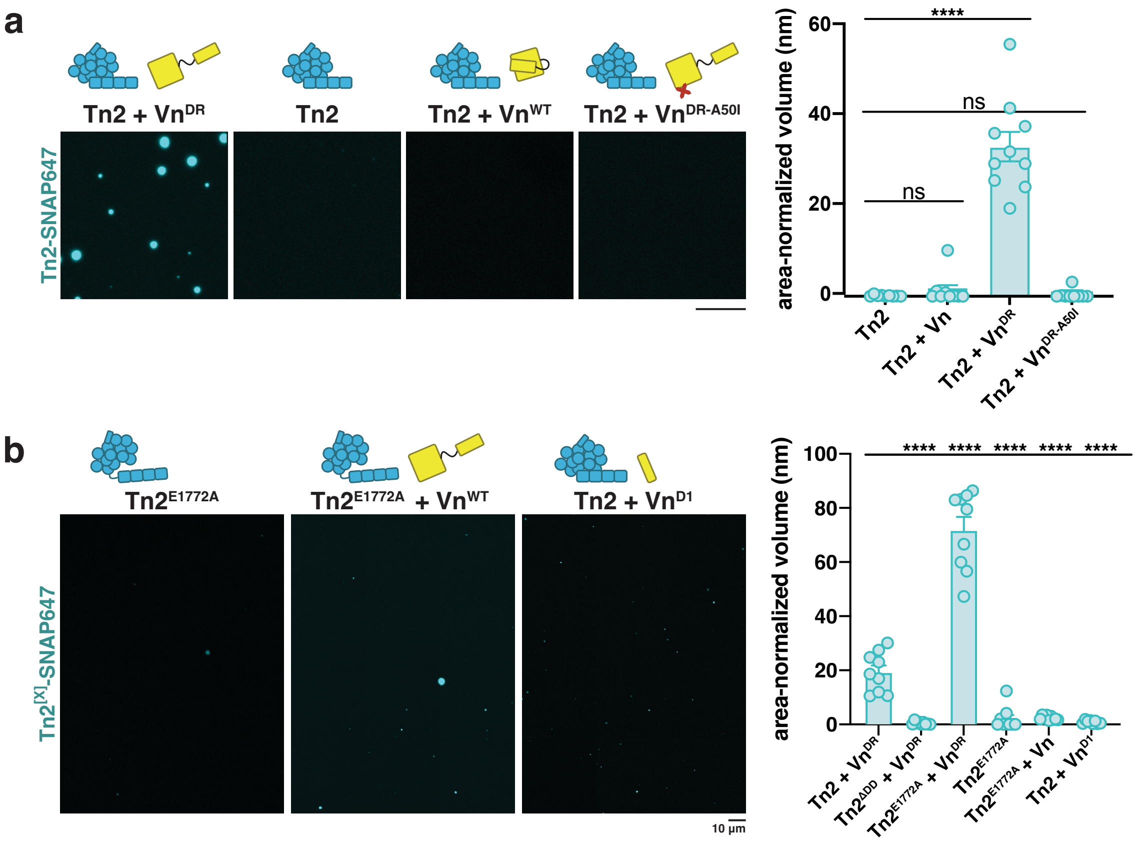

Supplementary Figure S7. Phase separation of talin requires full-length, deregulated vinculin. a Additional conditions for Fig. 1f and quantification of the volume of phase separated material from confocal z-stacks. Disrupting the talin-vinculin interaction in deregulated vinculin with the A50I mutation blocks phase separation of talin. Data are shown as mean values +/- SEM. For each condition, $n \geq 9$ regions were analysed from 3 different samples. **** represents $p < 0.0001$. **b** Additional conditions for Fig. 1g and quantification of the volume of phase separated material from confocal z-stacks. Phase separation of talin with de-regulated vinculin is enhanced when autoinhibition of talin is weakened by the E1772A mutation. The Vn^{D1} domain, which contains the talin binding site, is insufficient for the formation of talin condensates. Data are shown as mean values +/- SEM. For each condition, $n \geq 8$ regions were analysed from 3 different samples. **** represents $p < 0.0001$. Scale bar is 10 µm. Error bars represent standard error. Statistical test performed is an unpaired two-tailed t-test.

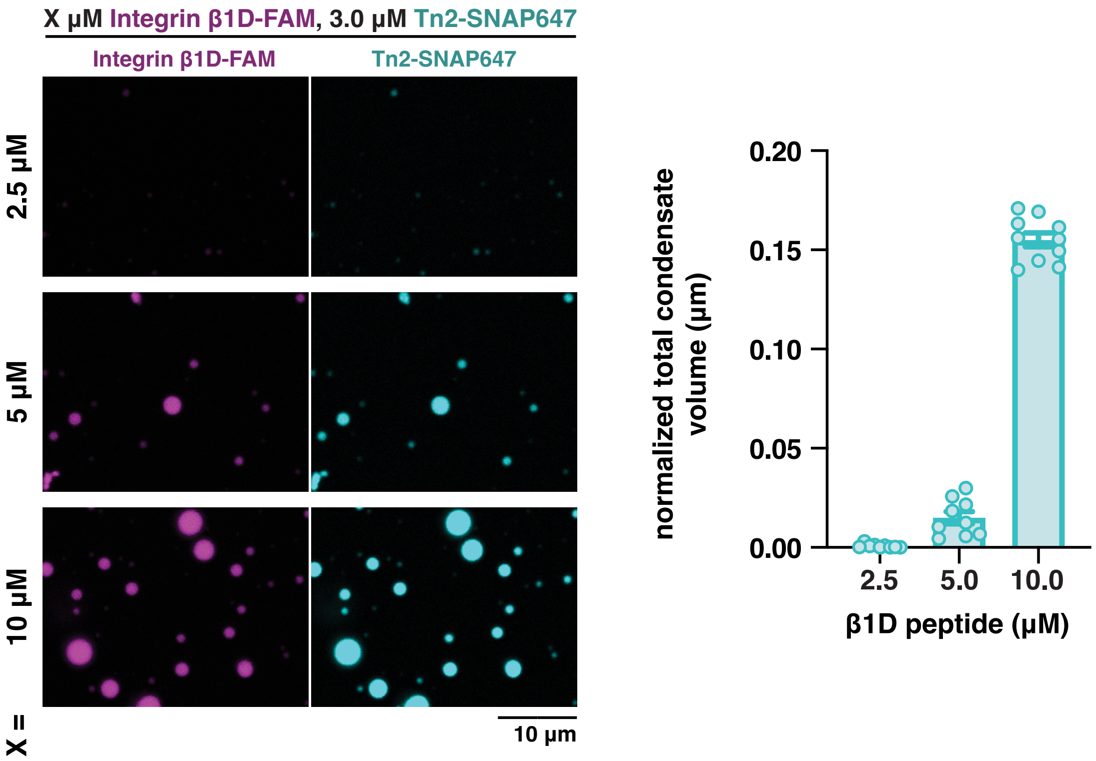

Supplementary Figure S8. Phase separation of talin with β1D peptide is concentration dependent. Representative confocal microscopy images of Tn2-β1D droplets formed at increasing concentrations of β1D integrin peptide. The amount of total condensate volume is quantified for the varying concentrations from confocal z-stacks. Tn2 was mixed with β1D integrin peptide and incubated for 1 hour before imaging. Data are shown as mean values +/- SEM. For each condition, n ≥ 9 regions were analysed from 3 different samples. Scale bar is 10 μm.

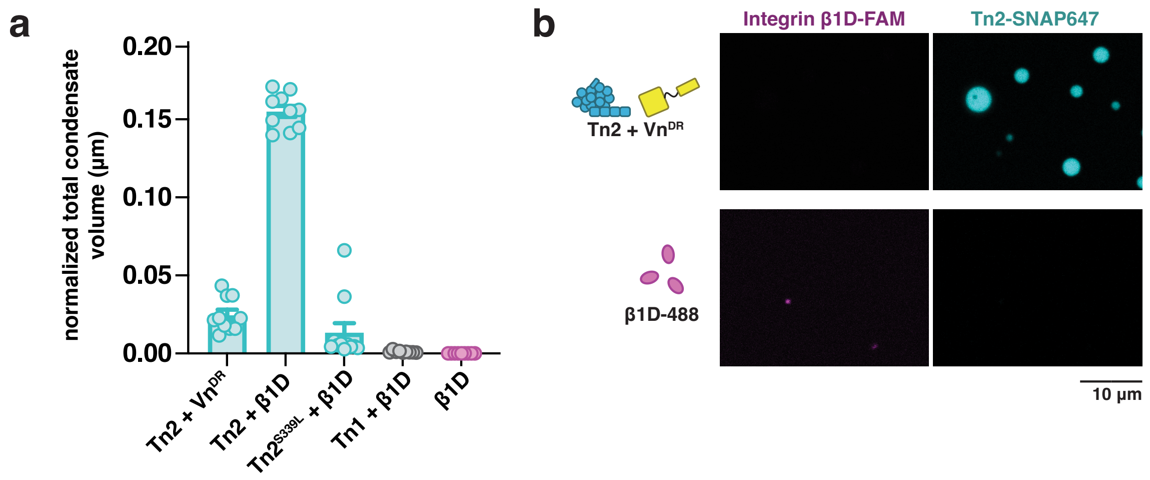

Supplementary Figure S9. Integrin β1D drives phase separation of Tn2, not Tn1. Additional conditions and quantification corresponding to Fig. 2b. **a** Tn2-β1D droplet formation requires the residue responsible for Tn2's higher affinity for the β1D integrin receptor, indicating a specific effect. Additionally, Tn1 does not form droplets with the β1D peptide, consistent with the importance of the talin2 S339L residue. 3 µM TnX was mixed with 10 µM β1D integrin peptide and incubated for 1 hour before imaging. Data are shown as mean values +/- SEM. For each condition, n ≥ 9 regions were analysed from 3 different samples. **b** Additional images correspond to Tn2 + Vn^{DR} (top) and β1D alone (bottom). Scale bar is 10 µm.

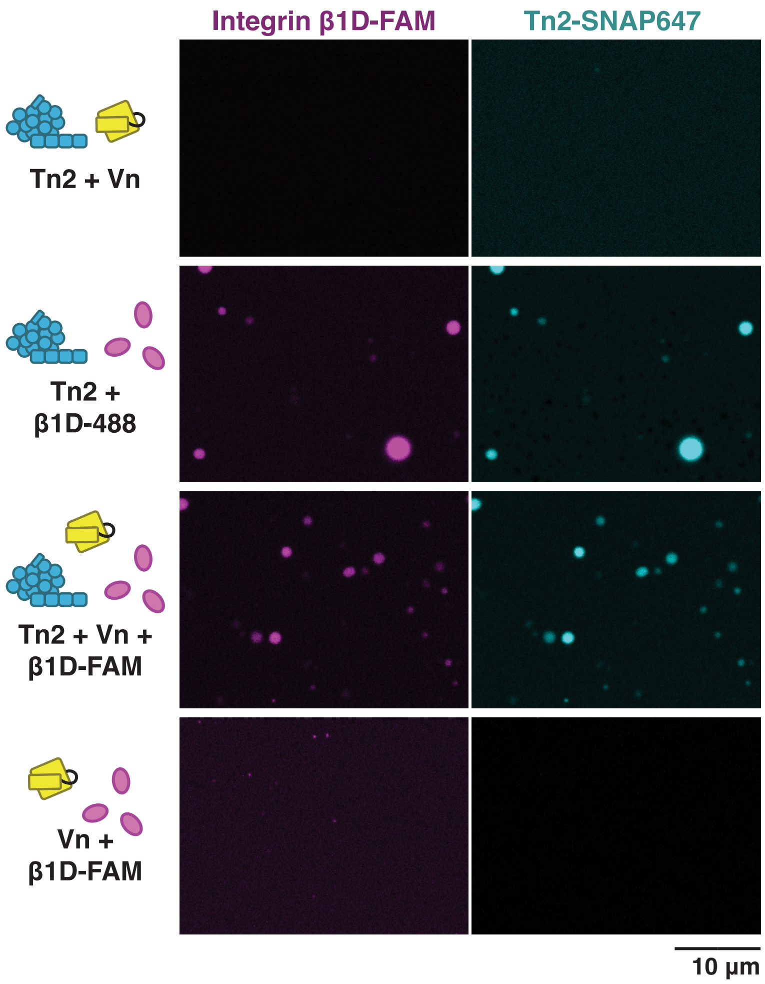

Supplementary Figure S10. Vinculin reduces phase separation of Tn2-β1D, and does not phase separate with β1D alone. Representative images for data displayed in Fig. 2d. 3 μM Tn2 and/or 3 μM Vn were mixed with 10 μM β1D integrin peptide and incubated for 1 hour before imaging. Scale bar is indicated in the figure. Scale bar is 10 μm.

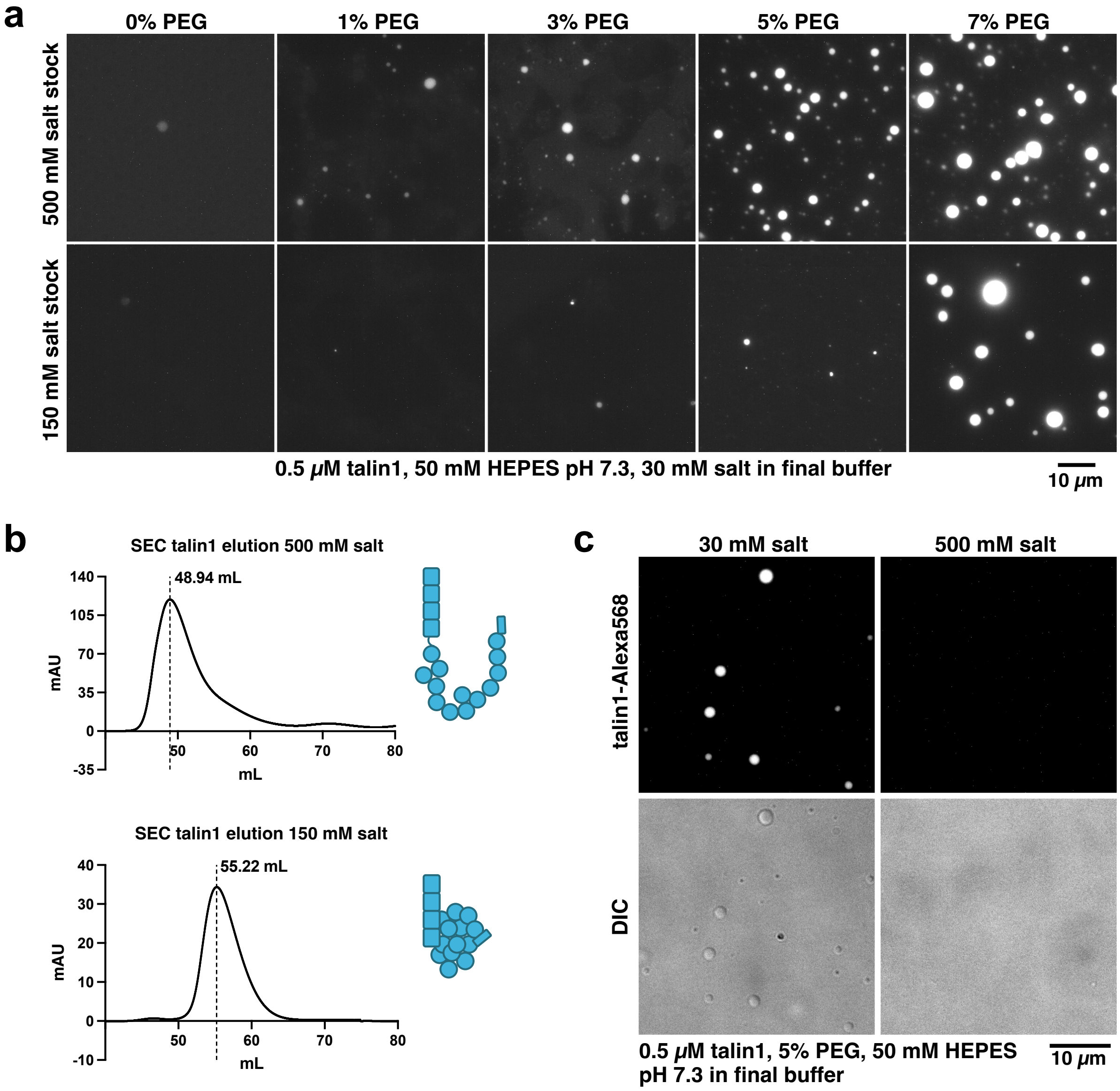

Supplementary Figure S11. Talin1 phase separation can be induced by drastic reduction of salt in buffer. Previous study has shown with cryoEM (Dedden et al 2019) that 80% of talin1 exhibit open conformation in buffer with 500 mM salt (NaCl or KCl), while only 20% are open in buffer with 150 mM salt. **a** Upon dilution of talin1 pre-incubated at 500 mM salt (top panel) to low salt buffer (30 mM salt), phase separation occurs, relative to the amount of crowding agent (PEG) present. A similar trend is observed with talin incubated at 150 mM salt (bottom panel), although phase separation requires a much higher concentration of crowding agent, consistent with a smaller percentage of talin1 in an open conformation. **b** Talin1 elution from size-exclusion column with buffer containing different salt concentrations. With 500 mM salt, talin1 elutes earlier than with 150 mM salt, consistent with a more open, less globular conformation. **c** Talin1 condensates formed in the presence of crowding agent can be dissolved with high salt that disrupts protein-protein interaction.

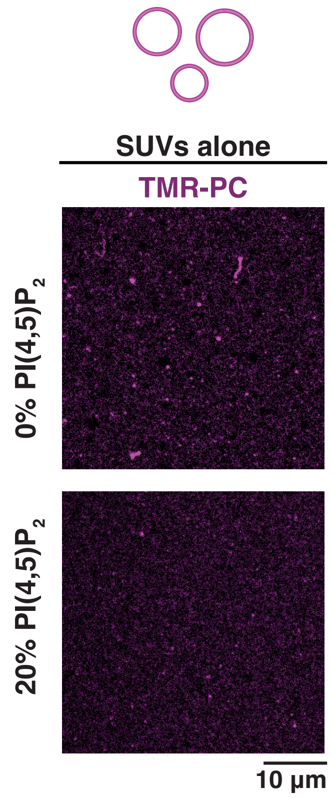

Supplementary Figure S12. Control images for vesicles alone. Under the same conditions as Fig. 3b without Tn2, SUVs are too small to be discernable. No droplets are observed for either lipid composition without additional proteins. Intensity has been increased relative to Fig. 3b in order to see lipid signal. Liposomes contained 74.75-X% DOPC, 15% DOPE, 10% DOPS, X% PI(4,5)P_2, 0.25% TopFluor TMR-PI(4,5)P_2. Scale bar is 10 μm. All lipid-based experiments were carried out in the following buffer (10 mM HEPES pH 7.5, 100 mM NaCl).

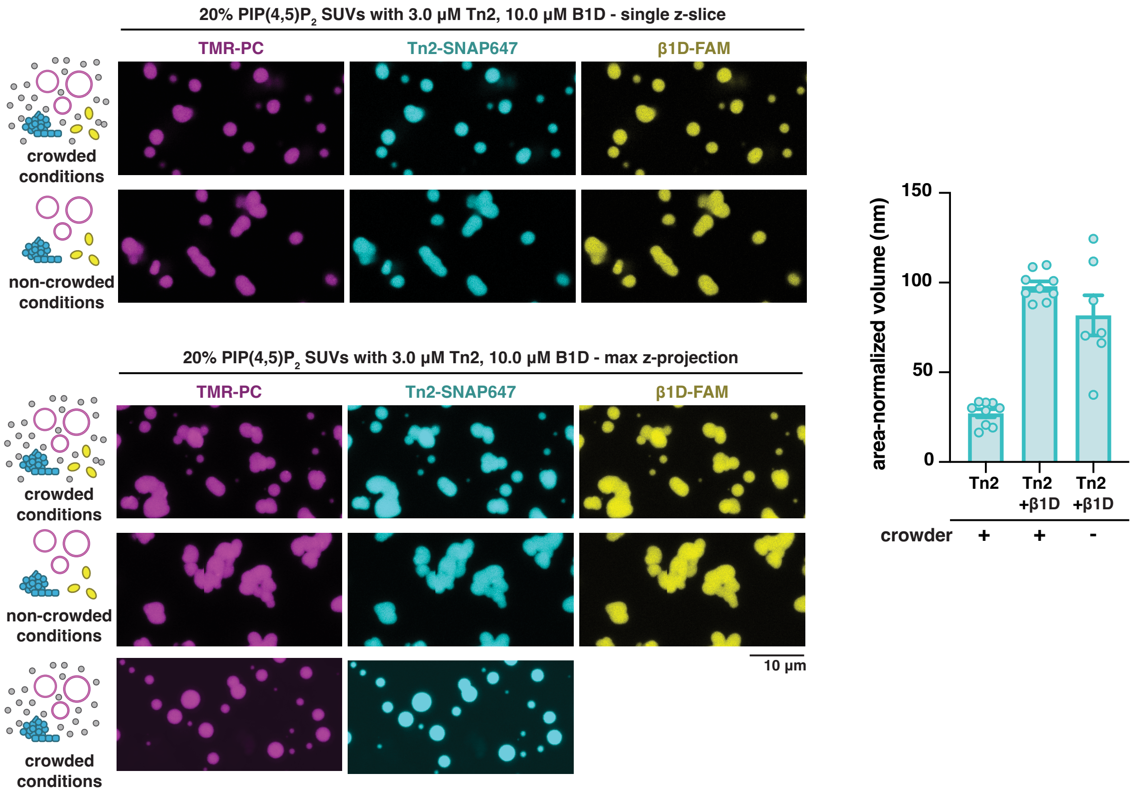

Supplementary Figure S13. Tn2-β1D-SUV droplets form even in the absence of crowder. a Single slice images of Tn2-β1D-SUV droplets with or without methyl cellulose. **b** Max z-projections from z-stacks of Tn2-β1D-SUV droplets with or without methyl cellulose. Droplets formed in the presence of all three components are less spherical, presumably because they do not reform a spherical shape after fusing. Instead, the droplets take on a pearls-on-a-string appearance, suggesting a gel-like condensate. The amount of phase separated material is much higher in the presence of both SUVs and β1D, regardless of whether crowder is included. Data are shown as mean values +/- SEM. For each condition, n ≥ 7 regions were analysed from 3 different samples. Liposomes contained 54.75% DOPC, 15% DOPE, 10% DOPS, 20% PI(4,5)P_2, 0.25% TopFluor TMR-PI(4,5)P_2. All lipid-based experiments were carried out in our lipid buffer (10 mM HEPES pH 7.5, 100 mM NaCl), either with 0.25% methyl cellulose or without crowding reagent. Scale bar is 10 µm.

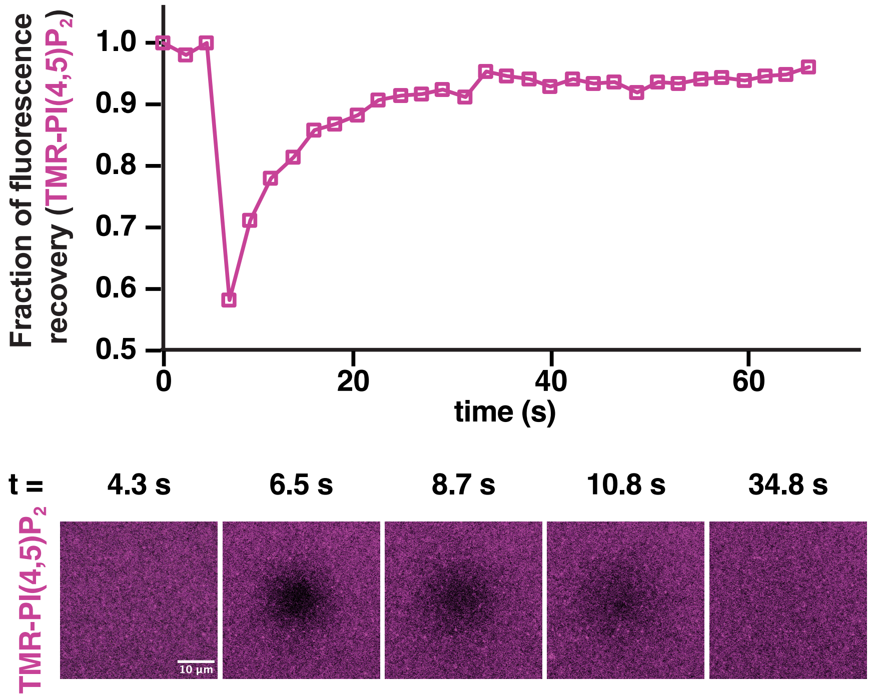

Supplementary Figure S14. Fluorescence recovery after photobleaching (FRAP) experiment on PI(4,5)P$_2$-containing supported lipid bilayer (SLB). We performed a FRAP experiment on an SLB containing 5% PI(4,5)P$_2$. We observe a rapid recovery of the fluorescently labeled PI(4,5)P2 in the bleached region, wich shows that the lipids in the bilayer are in a fluid state. Due to the rapid diffusion of the lipid molecules, the boundaries of the bleached region are blurry in our images, even in the first frame after bleaching (t = 6.5 s). Shown is data from a single FRAP experiment.

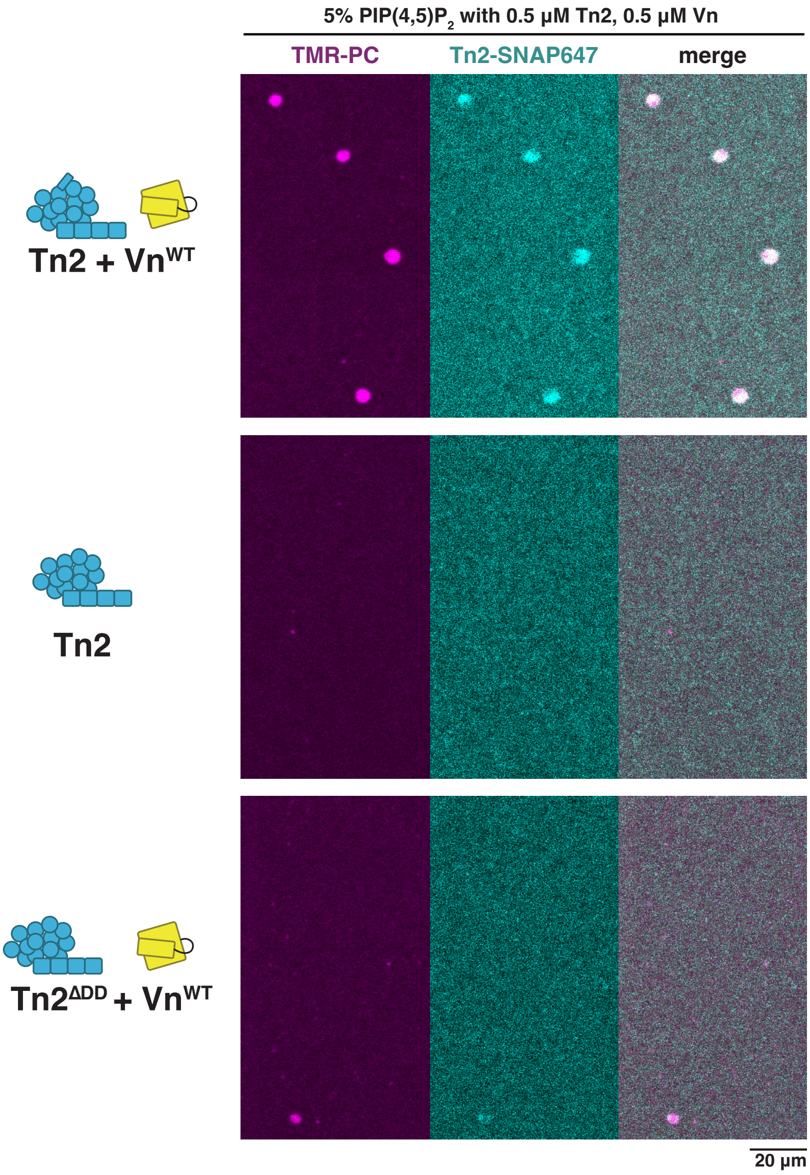

Supplementary Figure S15. Tn2-PI(4,5)P_2 clusters on SLBs require Vn and the Tn2 dimerization domain. Despite Tn2 binding to the membrane, no clusters are observed in the absence of Vn or Tn2$^{\Delta DD}$ with Vn. Proteins were mixed and added to the SLB immediately after bilayer formation. Samples were incubated for 1hour before imaging. SLBs were made from liposomes containing 69.75% DOPC, 15% DOPE, 10% DOPS, 5% PI(4,5)P_2, 0.25% TopFluor® TMR PI(4,5)P_2. Scale bar is 20 μm.

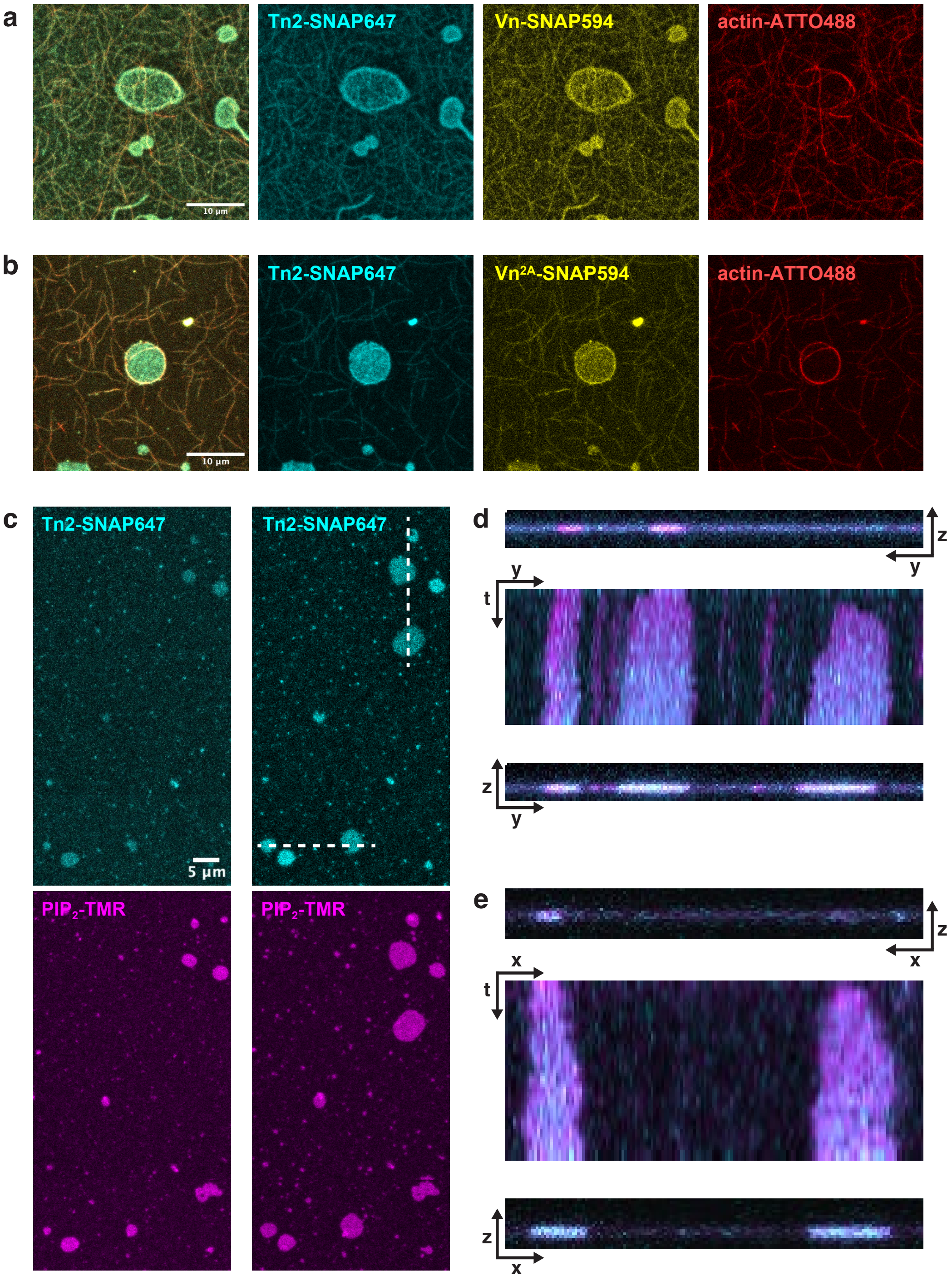

Supplementary Figure S16. Contents and dynamic behavior of protein clusters on SLBs. a, **b** Experiment on SLBs with labeled vinculin. 8% PI(4,5)P_2 with 1 μM Tn2, 1 μM Vn and 1 μM actin, supplemented with fluorescently labeled variants of each protein: Tn2-SNAP647, Vn-SNAP594, actin-ATTO488. The SNAP tag on vinculin appeared to affect condensate dynamics, hence we generally omitted using it in our experiments on SLBs. The images shown here however demonstrate that, as expected, vinculin co-localizes with talin in 2D condensates on membranes. **a** Experiment with wild-type vinculin. **b** Experiment with Vn^{2A} mutant, as described in Kelley et al. 2020. **c** - **e** Dynamic behavior of protein clusters. Experiment on SLBs containing 8% PI(4,5)P_2 with 1 μM Tn2, 1 μM Vn and 1 μM actin, supplemented with Tn2-SNAP647 and

PI(4,5)P_2-TMR. **c** Left two panels shows experiment at an earlier time compared to right two panels. Difference in time is 30 min. **d** and **e** show side views of selected areas in **c. d** shows side view of area surrounding the white dotted line in the top right corner. **e** shows cross section along white dotted in bottom left corner in **c**. Both **d** and **e** show 3 images each. First image is side view at t = 0. The second image is a kymograph along the membrane. The third image is side view at t = 30 min.

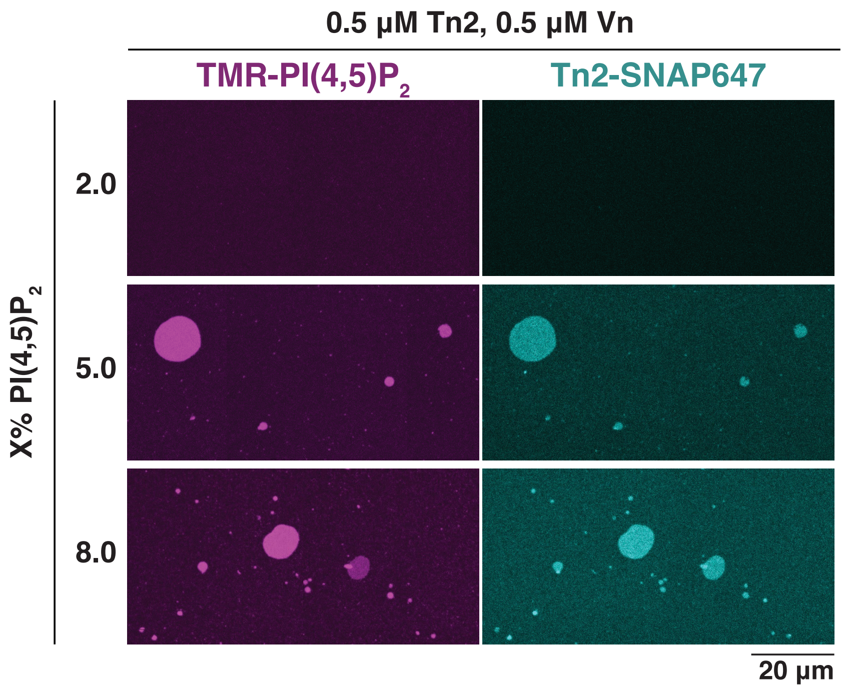

Supplementary Figure S17. PI(4,5)P_2 drives formation of Tn2 clusters on SLBs. Tn2 forms clusters on 5% and 8% PI(4,5)P_2 SLBs, but not on those with only 2% PI(4,5)P_2, suggesting there is a threshold for cluster formation dependent on membrane composition. As Tn2 membrane binding is dependent on PI(4,5)P_2 levels, this suggests that a minimum density of Tn2 molecules on the membrane surface is required for cluster formation. Proteins were mixed and added to the SLB immediately after bilayer formation. Samples were incubated for 1 hour before imaging. Representative images are from experiments done in triplicate. SLBs were made from liposomes containing (74.75-X)% DOPC, 15% DOPE, 10% DOPS, X% PI(4,5)P_2, 0.25% TopFluor® TMR PI(4,5)P_2. All lipid-based experiments were carried out in the following buffer (10 mM HEPES pH 7.5, 100 mM NaCl). Scale bar is 20 µm.

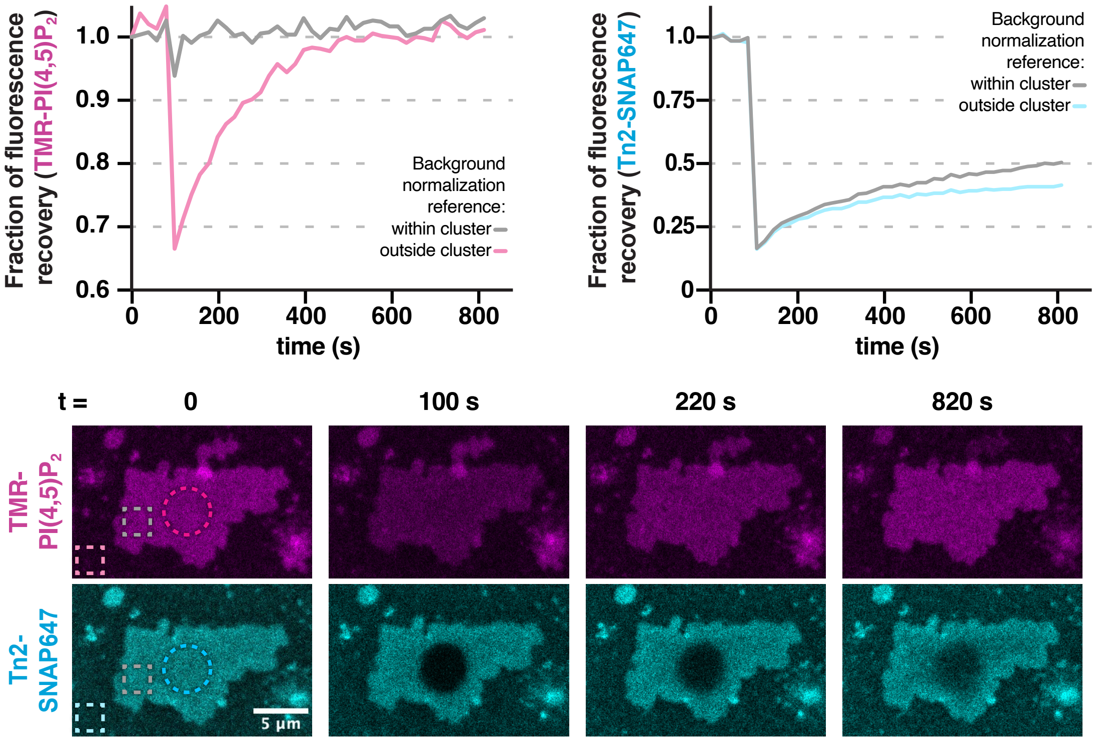

Supplementary Figure S18. FRAP experiment on region within Tn2-Vn-PIP$_2$ clusters. Experiment on SLB with 8% PIP$_2$, 1µM Tn2, 1µM Vn and 1µM actin. We performed a FRAP experiment on protein-lipid clusters on an SLBs in both the PIP$_2$ and the Tn2 channel (FRAP region: magenta/blue circle). We see different recovery rates for the two channels, similar to Figure 3e. We also see different rates when comparing molecular exchange within the dense phase and molecular exchange between the dense phase and the dilute phase/volume above the membrane. We look into this, by using different reference areas for background correction. By using an area within the condensate (gray area) for background correction, the resulting curve (gray curves) tells us about exchange within the condensate. By using an area outside the condensate (light blue/magenta area) for background correction, the resulting curve (light blue/magenta curves) tells us about recovery through molecules from outside the cluster. The difference is particularly noticeable for the recovery of PIP$_2$: Exchange within the cluster is relatively fast, so that with the low time resolution of this data (1 frame/20 seconds), the bleached area is not visible and the signal within the cluster is already homogenous in the first frame after bleaching. Hence a dip in fluorescence does not exist in the gray curve for PIP$_2$. Recovery of the cluster itself (i.e. exchange with the dilute phase), is much slower, but fluorescence still fully recovers after a few minutes (light magenta curve). The two plots for Tn2 recovery are much more similar to each other. Given the very different nature of the molecules, this discrepancy between PIP$_2$ and Tn2 is not surprising. PIP$_2$ is entirely restricted to a 2D membrane, while Tn2 can exchange with the solution above. In fact, the observation that the curves for dilute phase-normalized Tn2 signal and cluster-normalized Tn2 signal are similar, could lead to the conclusion that recovery mostly happens through exchange with the solution above. However this does not agree with our microscopy images in which 2-dimensional

gradients at the boundaries of the bleached region and at cluster boundaries can be observed, indicating that Tn2 also diffuses laterally on the membrane. It should also be noted that generally Tn2 recovery seems to be much slower compared to the recovery of the lipids underneath and compared to Tn2 recovery on SLBs without Vn (i.e. without condensate formation) (Figure S19), indicating that interactions within the dense phase reduce diffusivity of the proteins. Shown is data from a single FRAP experiment.

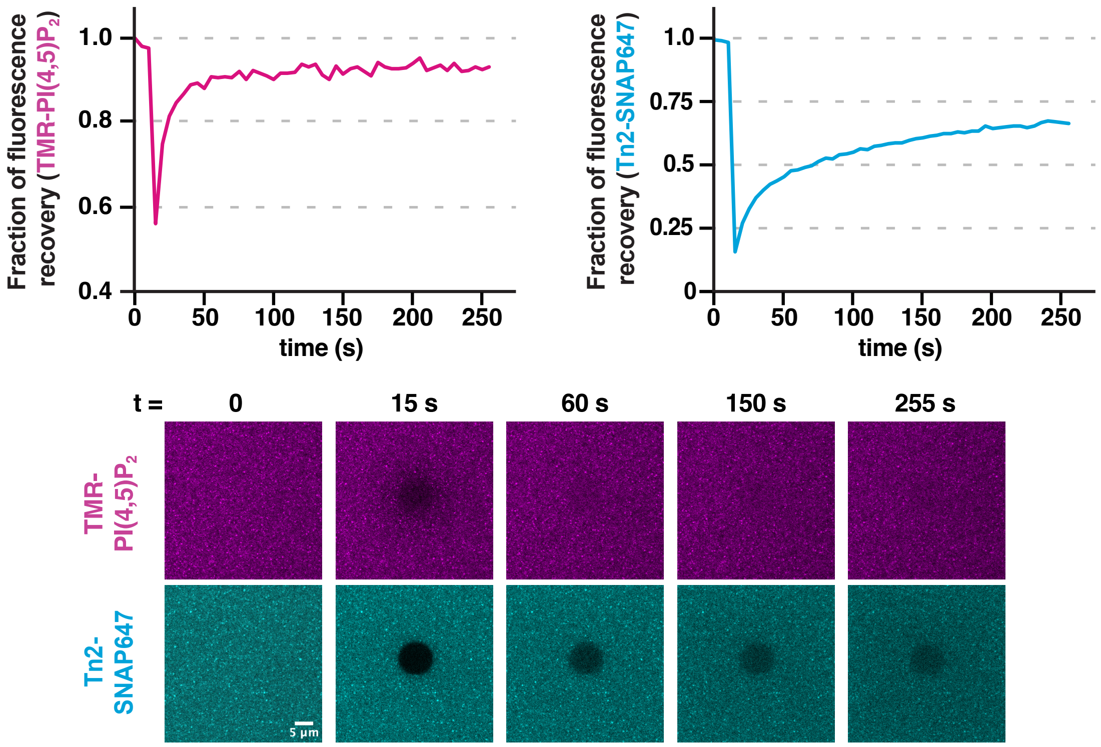

Supplementary Figure S19. FRAP experiment of membrane-bound Tn2. Experiment on SLB with 5% PIP_2, 0.5 µM Tn2. Tn2 binds to PIP_2-containing membranes, but does not form clusters without the addition of Vn (see Figure S15). The FRAP experiment shows that in this state, Tn2 is highly diffusive on the membrane. Shown is data from a single FRAP experiment.

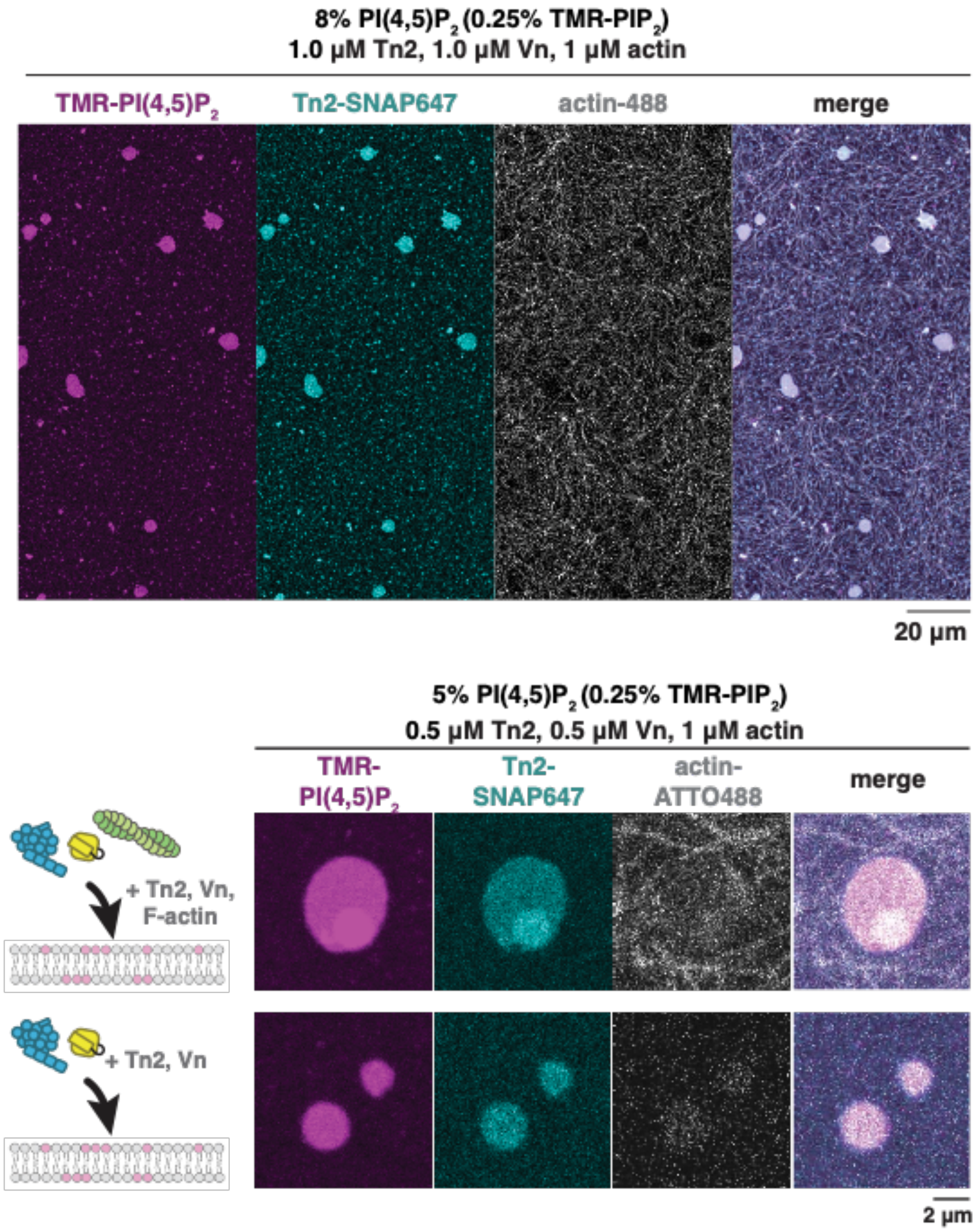

Supplementary Figure S20. Actin is not recruited to Tn2-PI(4,5)P$_2$ clusters. F-actin is recruited to the membrane surface in the presence of Tn2 and Vn, but does not become enriched within Tn2-PI(4,5)P$_2$ clusters. Actin was polymerized at RT for 30 minutes, then mixed with Tn,Vn at the indicated concentrations and incubated for 1 hr before imaging. A field-of-view image indicates that actin filaments localize around Tn2-PI(4,5)P$_2$ on the membrane, while the zoomed in view confirms that actin is not enriched within the clusters. Scale bars are indicated in the figure. SLBs were made from liposomes containing 66.75% DOPC, 15% DOPE, 10% DOPS, 8% PI(4,5)P$_2$, 0.25% TopFluor® TMR PI(4,5)P$_2$.

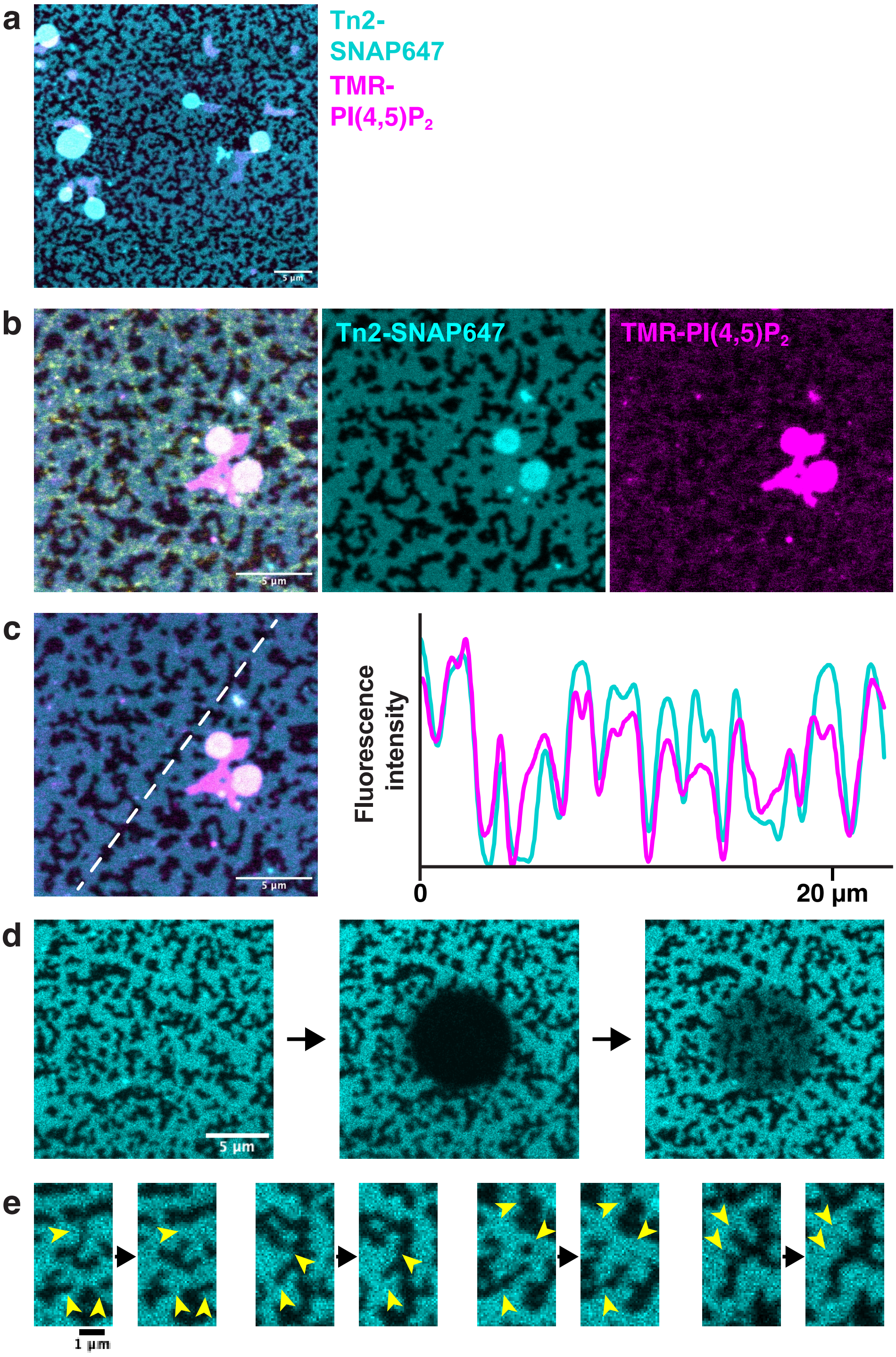

Supplementary Figure S21. Spinodal decomposition of Tn2 on PIP_2-SLBs. We occasionally observed patterns of Tn2 on the PIP_2-containing SLB that appear to form through spinodal decomposition. Spinodal decomposition is a distinct type of phase separation, which occurs due to thermodynamic fluctuations, without the need for nucleation, and results in characteristic patterns. Reproducability of these results was low. It is likely that there is a dependence on factors such as changes in temperature, that we, thus far, have not controlled carefully enough to understand their effects. However, we think it is worth reporting this data here, as it a very direct indicator of liquid-liquid phase separation taking place. Experiments shown here are performed with 1 µM talin2, 1 µM Vn and 1 µM actin. **a** Image with lower zoom and merged channels for Tn2 and PIP_2. **b**

Image with higher zoom compared to (A). First panel shows merged channels of Tn2-SNAP647, TMR-PIP_2 and actin-ATTO488, followed by single channel images for Tn2-SNAP647 and TMR-PIP_2. Intensity and contrast of PIP_2 signal in single-channel image (right) is increased compared to merged image (left). **c** The plot (right) shows the intensity profile along the white dotted line in the image on the left. The PIP_2 lipids did not form clusters of high density that co-localize with the 2D Tn condensate above as clearly as for the condensates formed through nucleation (Figure 3d - g). This can be explained by the increased area of dense phase when compared to nucleated condensates, in combination with the limited reservoir of PIP_2 compared to Tn2 (the latter being available from the solution above). However, when adjusting intensity and contrast, a slightly increased concentration of PIP_2 could still be identified to co-localize with the Tn2 patterns, as shown in the intensity profile plot. **d** FRAP experiment showing partial fluorescence recovery after 150 seconds. Fluorescence signal for Tn-SNAP647 is shown. **e** Patterns change dynamically and show coarsening. 4 different examples with two frames each which are 165 seconds apart. Arrows highlight notable features that change. Fluorescence signal for Tn-SNAP647 is shown.

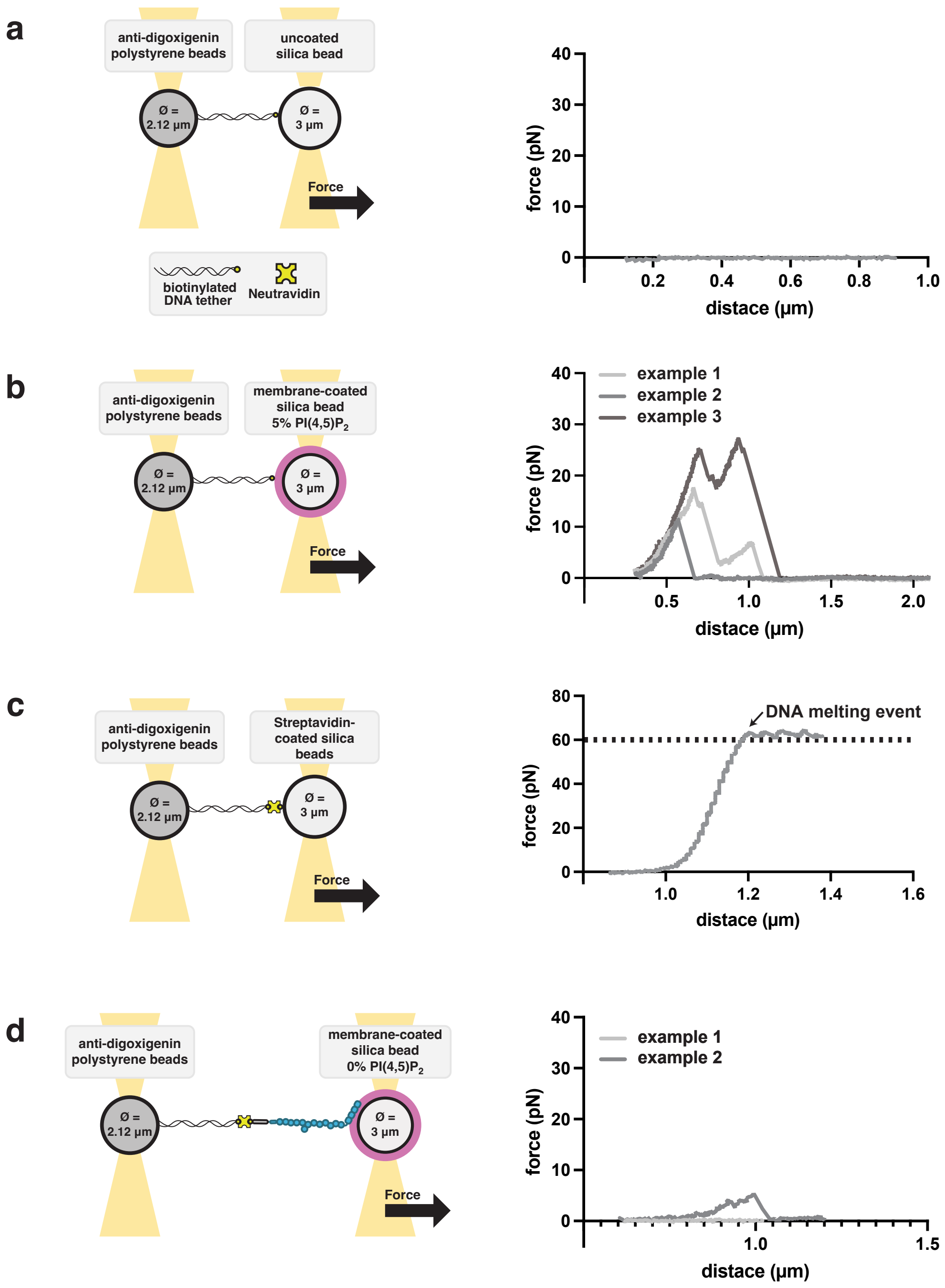

Supplementary Figure S22. Tethering controls. a Tethers were not detected between anti-DIG beads with biotinylated DNA strands and uncoated silicon beads. **b** Some tethers were detected between anti-DIG beads with biotinylated DNA strands and silica beads coated with 5%PI(4,5)P_2 lipid membranes, but at a lower frequency than in the presence of talin. Additionally, the distance at which rupture occurred distinguished these tethers from true talin-membrane interactions (see Figure S24). Three example curves are shown, rupturing at various forces, but all below 1um distance. **c** A streptavidin-coated silica bead is used to demonstrate a stereotypical force curve for a single DNA strand between two beads, and the

melting event that occurs at a force of 60 pN. **d** Control experiment with a DNA-Tn2 tether and a membrane-coated silica bead, however without PIP_2 in the lipid membrane.

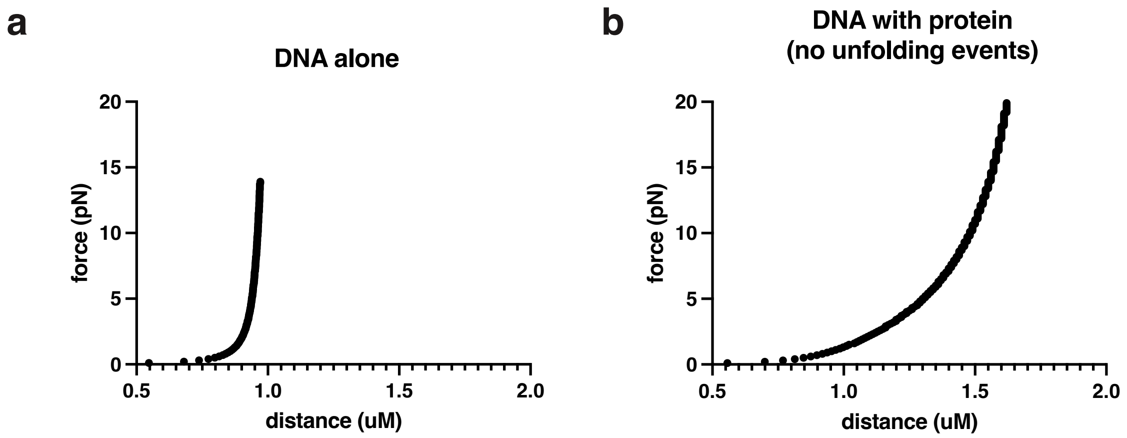

Supplementary Figure S23. Worm-like chain models for DNA force curves. a DNA alone **b** DNA-protein force curve, without unfolding events.

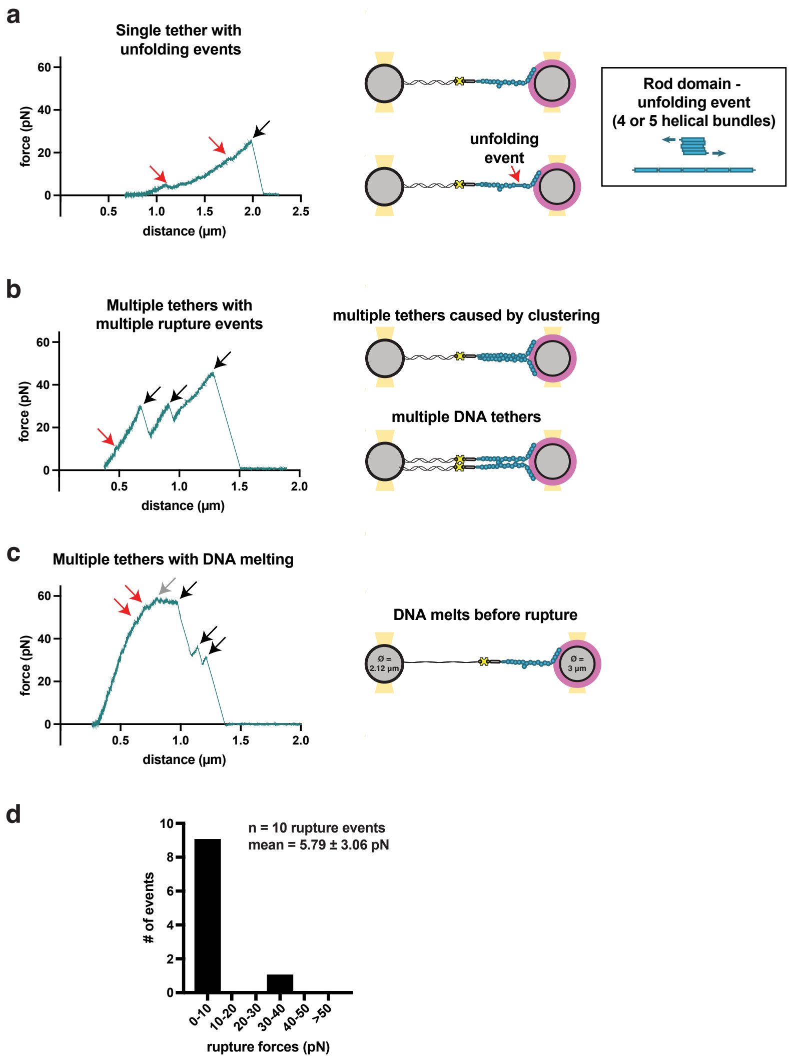

Supplementary Figure S24. Examples of Tn2 tethers with 5% PIP_2. Individual examples of force curves obtained with Tn2 bound to 5% $PI(4,5)P_2$-containing membrane-coated beads. **a** A single DNA-Tn2 tether, with multiple Tn2 unfolding events (red arrow) and a rupture event at a distance of 2 µm (black arrow). The inset figure shows a model of an individual helical bundle within the talin rod domain. These helical bundles are made up of either 4 or 5 helices. When placed under tension, as shown here, the helical bundle unfolds, taking on an extended, linear conformation. **b** An example of multiple Tn2-membrane tethers pulled simultaneously, and rupturing sequentially. The steep initial increase in force is indicative of multiple tethers, which could be due to multiple Tn2-DNA tethers, or due to interactions between the membrane-bound talin molecules themselves. **c** An example of multiple Tn2-membrane tethers which withstand more than 60 pN of force, at which point the DNA strand melts (gray arrow). This is an example of a connection too strong to be included in analysis. Rupture events (black arrows) can then been observerd after the DNA melting event. **d** Rupture force distribution for interaction of Tn2 with $PI(4,5)P_2$ bilayer. There were very few measurable force curves for Tn2 using this experimental set up, making the data difficult to interpret.

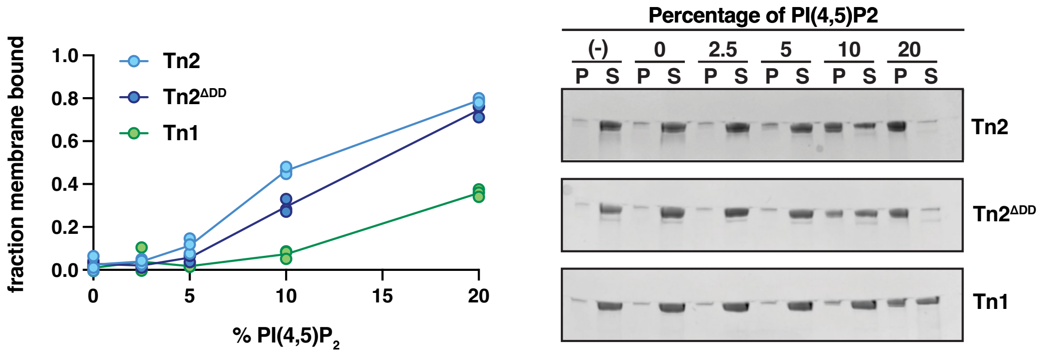

Supplementary Figure S25. Tn2, Tn1, and Tn2$^{\Delta DD}$ membrane binding depends on PI(4,5)P_2 levels. Tn2 and Tn2$^{\Delta DD}$ bind at similar levels, while Tn1 cosediments at lower levels even with the highest amount of PI(4,5)P_2. This is consistent with published reports that Tn1 has a lower affinity for membrane binding. The graph shows the fraction of total protein present in the pellet after centrifugation with vesicles with the indicated percentage of PI(4,5)P_2. Each condition was carried out in triplicate in buffer of 20 mM HEPES, pH 7.5 and 100 mM NaCl. Representative SDS-PAGE gels are shown on the right, with pellet (P) and supernatant (S) samples for each protein at each lipid composition, including a no lipid control sample (-). Liposomes contained 75-X% DOPC, 15% DOPE, 10% DOPS, X% PI(4,5)P_2. Each condition represents data from n = 3 independent incubations. Lines connect mean values.
