# Supplementary material for: Membrane-induced 2D phase separation of the focal adhesion protein talin
Source: Nat Commun. 2024 Jun 11;15:4986. doi: 10.1038/s41467-024-49222-z (PMC11166923; doi:10.1038/s41467-024-49222-z)
Supplement: Supplementary file 3 — Description of Supplementary Information [file 41467_2024_49222_MOESM3_ESM.docx]

**Description of Additional Supplementary Files**

File Name: Supplementary Movie 1

Description: Confocal microscopy time lapse video showing repeated fusion of Tn2-VnDR droplets. Same conditions as in Figure 1C-E, i.e. 3 μm VnDR and 3 μm Tn2 doped with Tn2-SNAP647. Most droplets are likely forming in the solution above the focal plane and then settle onto the glass slide, thus suddenly appearing in the focal plane and leading to more frequent observations of fusion events.

File Name: Supplementary Movie 2

Description: Fluorescence recovery after photobleaching (FRAP) experiment of a region within a Tn2 condensate. Experiment with 2 μM Tn2, 2 μM VnDR and 0.25% methyl cellulose.
